# Mcm2 predicts recurrence hazard in stage Ta/T1 bladder cancer more accurately than CK20, Ki67 and histological grade

**DOI:** 10.1038/sj.bjc.6603784

**Published:** 2007-05-15

**Authors:** M Burger, S Denzinger, A Hartmann, W-F Wieland, R Stoehr, E C Obermann

**Affiliations:** 1 Department of Urology, Landshuterstr. 65, D-93053 Regensburg, Franz-Josef-Strauss-Allee 11, D-93053 Regensburg of the University of Regensburg, Regensburg, Germany; 2 The Institute of Pathology, Franz-Josef-Strauss-Allee 11, D-93053 Regensburg of the University of Regensburg, Regensburg, Germany

**Keywords:** Mcm2, Ki67, CK20, bladder cancer

## Abstract

Stage Ta/T1 urothelial carcinoma of the bladder (Ta/T1 BC) has a marked tendency to recur. Besides histopathology, markers such as CK20 expression and proliferation index (Ki67) have been shown to predict its clinical course. The replication-licensing factor minichromosome maintenance protein 2 (Mcm2) is a marker of proliferative potential shown to be a promising prognostic marker in various malignancies. The aim of the present study was to evaluate the prognostic value of Mcm2 in comparison to stage, grade, CK20 and Ki67. Initial sporadic Ta/T1 BC (*n*=71) were evaluated for their expression of CK20, Ki67 and Mcm2 by immunohistochemistry and tissue microarray technology. Prognostic power was analysed by univariate and multivariate Cox regression model for tumour recurrence rate. Median follow-up period was 39 months. A total of 35% patients experienced recurrence. While CK20 was not predictive, grade, Ki67 and Mcm2 were significantly related to recurrence rate in univariate Cox regression model. Only grade (HR 2.37; 95% CI 1.24–4.51; *P*=0.009) and Mcm2 expression with a cutoff ⩾40% (HR 5.81; 95% CI 2.41–14.00; *P*<0.001) were independent predictors of recurrence rate in multivariate Cox regression analysis. In addition to grade, expression of Mcm2 is an independent predictor of recurrence in Ta/T1 BC.

Urothelial carcinoma of the bladder is the fifth most common cancer. While it is mostly non-muscle-invasive (i.e. pTa and pT1) at initial presentation, up to 70% of patients experience recurrent disease and up to 15% will progress to muscle-invasive BC (Millan-Rodriguez *et al*, 2000). While cases with favourable biological behaviour are subject to intense and frequent surveillance without need, patients with high-risk tumours are depending on close monitoring. Thus reliable prognostic parameters differentiating high- and low-risk patients with stage Ta/T1 urothelial carcinoma of the bladder (Ta/T1 BC) might enable individual risk adapted follow-up regimes. Despite high interobserver variation, histological grading is the most commonly used predictor of prognosis in Ta/T1 BC (Tosoni *et al*, 2000). Two well-established markers of recurrence are MIB-1 and CK20. CK20 is normally expressed by umbrella cells. CK20 dedifferentiation (e.g. expression in the entire urothelium or complete expression loss) has been related to disease recurrence in Ta/T1 BC (Harnden *et al*, 1999). The proliferation index MIB-1, an antibody detecting the nuclear antigen Ki67 expressed by dividing cells, has been linked to progression (van Rhijn *et al*, 2003; Lopez-Beltran *et al*, 2004). Recently, analysis of minichromosome maintenance protein (Mcm) expression has been demonstrated to have prognostic value in prostate and renal cell cancer and B-cell lymphoma (Meng *et al*, 2001, Rodins *et al*, 2002, Obermann *et al*, 2005). Minichromosome maintenance proteins are presumed to regulate replication by cyclical DNA unwinding and to be highly specific for proliferation (Obermann *et al*, 2005). Minichromosome maintenance protein 5 has been shown to detect bladder cancer reliably in voided urine (Stoeber *et al*, 1999). To our knowledge there are only two studies investigating the expression of Mcm2 in bladder cancer. Krüger *et al* (2003) showed Mcm2 expression to be the only parameter predicting progression in 52 pT1 tumours. Korkolopoulou *et al* (2005) found Mcm2 and Mcm5 to indicate adverse outcome in 65 muscle invasive bladder cancers. The aim of this study was to evaluate the prognostic value of CK20 and Ki67 and to evaluate their relation to Mcm2 in Ta/T1 BC randomly chosen from a consecutive and unselected series.

## MATERIALS AND METHODS

### Patients

The study was approved by Institutional Review Board of the University of Regensburg and all patients gave informed consent. From the databank of the Department of Urology of the University of Regensburg containing 1200 initial Ta/T1 BC undergoing transurethral resection of the bladder (TURB) from 1985 to 2005, 100 patients with initial Ta/T1 BC were chosen by random generator and samples retrieved from the Department of Pathology. All tumours were fixed in formalin and embedded in paraffin. Clinical charts were reviewed and follow-up data were collected. All slides were reviewed by one uropathologist (AH). Tumour stage and grade were assigned according to the TNM (UICC, 2002) and WHO classification of malignant tumours of the urinary tract (Mostofi *et al*, 1973). All patients underwent control TURB 4–6 weeks after the initial TURB. Adjuvant therapy and follow-up procedure were initiated as reflected by appropriate guidelines (Oosterlinck *et al*, 2002).

### Tissue microarray and immunohistochemistry

**Figure 1:**
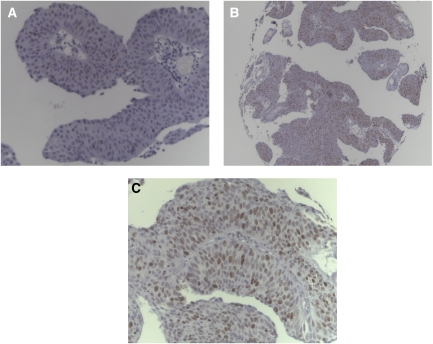
(**A**–**C**) Minichromosome maintenance protein 2 expression in a non-invasive papillary bladder cancer (pTa, low grade) showing levels of <20% (**A** × 200) 60% (**B** × 50 and **C** × 200).

Tissue microarrays (TMA) were constructed (Klopocki *et al*, 2004) and expression of Mcm2, Ki67 and CK20 was evaluated by immunohistochemistry (IHC) using standard staining procedures as described previously (Harnden *et al*, 1999; Lopez-Beltran *et al*, 2004; Obermann *et al*, 2005). Antigen retrieval was carried out in a microwave (250 W for 30 min, in a citrate solution; pH 6.0). The sections were incubated with primary monoclonal antibodies for Mcm2 (mouse monoclonal, clone 46, BD Biosciences, San Jose, USA, dilution 1 : 3000), CK20 (clone IT-Ks 20.8; DAKO, Glostrup, Denmark; dilution 1 : 100) and Ki67 (mouse monoclonal, clone MIB-1, DAKO, Hamburg, Germany, dilution 1 : 50). Negative controls were obtained by omitting the primary antibody. Standard procedures were used for visualisation (ABC-Elite, Vector-Laboratories, Burlingame, CA, USA). Diaminobenzidine was used as chromogen. The slides were evaluated without knowledge of clinical data. Immunoreactivity for CK20 and Ki67 was scored according to previously validated criteria (Harnden *et al*, 1999; Lopez-Beltran *et al*, 2004). CK20 staining pattern was scored normal in cases with expression in superficial umbrella cells and absence in the intermediate or basal urothelial cell layers; diffuse immunostaining (>10%) or lack of CK20 expression was scored abnormal as described previously (Harnden *et al*, 1999). High Ki67 labeling index was defined if ⩾15% of the tumour cells were stained as described previously (Lopez-Beltran *et al*, 2004). Immunoreactivity to Mcm2 was evaluated in 20% steps (Figure 1).

### Statistical analysis

Statistical analyses were completed using SPSS version 12.0. (SPSS, Chicago, IL, USA). *P*-values <0.05 were considered significant. Contingency table analysis and two-sided Fisher's exact tests were used to study the statistical association between the groups. Significant differences concerning tumour recurrence rate were calculated using the Kaplan–Meier method and log-rank test. For the evaluation of tumour recurrence rate, patients were surveyed at the time of their last tumour-free clinical follow-up appointment. Univariate and multivariate Cox regression models concerning tumour recurrence rate were adjusted, testing the independent prognostic relevance of the parameters analysed.

## RESULTS

### Clinical data and histopathology

Only cases with follow-up information were included in the analysis. Follow-up data could be obtained from 71 out of 100 patients who were included in the analysis while 29 patients were omitted. The 29 cases dropping out were distributed evenly across pTa, pT1, all grades, CK20, Ki67 and Mcm2 expression. Median age at the time of diagnosis was 71 years (range: 52–94 years). The female proportion was 30% (*n*=21). A total of 90% (*n*=64) were in stage pTa and 10% (*n*=7) in pT1, respectively. Grade was distributed as follows: G1 43% (*n*=31), G2 46% (*n*=33) and G3 10% (*n*=7). Median follow-up was 39 months (range: 1–133 months). The median follow-up in cases with recurrence was 37 months (range: 4–97 months) and in cases without recurrence 40 months (range: 1–133 months). The use of adjuvant therapy with mitomycin and BCG was distributed evenly between cases with and without recurrence.

Recurring disease occurred in 35% (*n*=25) of patients. Progression to muscle invasive disease was noted in 6% (*n*=4) of cases. Thus progression risk was not statistically analysed due to small numbers.

### Parameters in relation to tumour recurrence

*Stage and grade:* While stage (pTa *vs* pT1; P=0.656) was not related to disease recurrence, grade was significantly correlated to tumour recurrence (*P*<0.002; Table 1).

*Ki67*: Immunohistochemistry for Ki67 revealed a high proliferation index (⩾15%) in 30% of cases. High proliferation index was significantly related to tumour recurrence (*P*<0.001; Table 1).

*CK20*: CK20 was abnormal as defined above in 60% of cases. There was no relation between the expression pattern of CK20 and disease recurrence (Table 1).

*Mcm2*: Mcm2 expression level was ⩾40% in 29% of cases. Using a cutoff level of 40%, there was a strong relation with tumour recurrence (*P*<0.001; Table 1).

### Predictive factors for recurrence rate in univariate analysis

**Figure 2:**
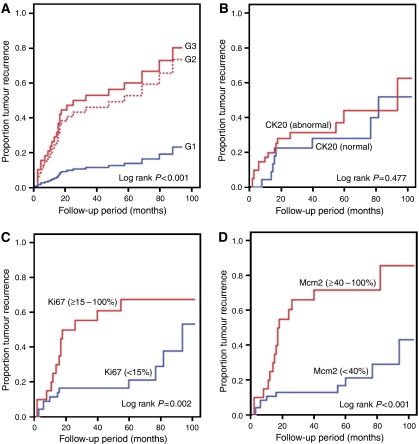
Kaplan–Meier analysis of tumour recurrence in relation to histopathological grade (**A**), CK20 (**B**), Ki67 (**C**) and Mcm2 (**D**).

Relationship by parameters studied and recurrence-free survival was evaluated by univariate Cox regression analysis. No significant relation to recurrence rate was found for stage, sex, age and CK20. In contrast, grade (*P*<0.001), Ki67 (*P*=0.002) and Mcm2 (*P*<0.001) were found to be prognostic of recurrence (Figure 2; Table 2).

### Predictive factors for recurrence rate in multivariate analysis

Multivariate Cox regression analysis was used to determine independent association of grade, stage, CK20, Ki67 and Mcm2 with recurrence. While stage, CK20 and Ki67 failed to demonstrate independence, grade (HR 2.37; 95% CI 1.24–4.51; *P*=0.009) and Mcm2 expression ⩾40% (HR 5.81; 95% CI 2.41–14.00; *P*<0.001) were independent predictors of recurrence (Table 2).

## DISCUSSION

The management of Ta/T1 BC of the bladder challenges urologists, as 70% of cases recur and 10–15% progress to invasive disease (Millan-Rodriguez *et al*, 2000). Completeness of excision has been recognised as a major factor for tumour recurrence (Brausi *et al*, 2002). Despite control TURB in all 71 Ta/T1 BC of the present series to enhance completeness of resection 35% experienced recurrences. Histopathological evaluation is the gold standard to predict clinical behaviour of Ta/T1 BC, despite recognised limitations such as high interobserver variability (Tosoni *et al*, 2000). Numerous markers have been evaluated to enhance the prognostic power of histopathological evaluation.

Two well-established markers are CK20 (Harnden *et al*, 1999) and Ki67 (van Rhijn *et al*, 2003; Lopez-Beltran *et al*, 2004). Ki67 is a commonly applied prognostic and diagnostic tool (van Rhijn *et al*, 2003; Lopez-Beltran *et al*, 2004), as the Ki67 protein is present in the nuclei of cells in the G_1_, S, and G_2_ phases of the cell cycle in dividing cells as well as in mitosis but not in the G_0_ phase of quiescent cells. Nevertheless it is unlikely that Ki67 reliably labels the entire proliferative cell fraction. Ki67 shows some variation in its expression in the G_1_ phase and may not be expressed in cells entering the G_1_ from G_0_. Its expression pattern also changes through the cell cycle so that the probability of detection by immunohistochemical staining may vary. In some tumours, Ki67 does not appear to label all proliferating cells (Verheijen *et al*, 1989; Hashimoto *et al*, 2004). Recently Mcm2 has been proposed as a valuable marker for tumour evaluation (Tachibana *et al*, 2005). In contrast to Ki67, Mcm2 is an essential factor for initiation of DNA replication in eukaryotic cells. Mcm family members are highly conserved replication initiation factors assembling with Cdt1 and Cdc6 proteins into pre-replicative complexes at origin recognition complexes during G_1_ phase (Maiorano *et al*, 2006), thereby licensing chromatin for replication in the subsequent S phase. Minichromosome maintenance proteins are present in all phases of the proliferative cell cycle but absent in quiescent or terminal differentiated cells and in cellular senescence. This defines the Mcm family as a novel class of proliferation marker (Maiorano *et al*, 2006). The detection of Mcm2–7 in tissues can be used to locate cells that have regained unscheduled proliferation activity which is one hallmark of cancer, and recent advances showed that Mcms could act as excellent markers for tumour evaluation (Tachibana *et al*, 2005).

We have evaluated CK20, Ki67 and Mcm2 in a consecutive and unselected series of Ta/T1 BC. While previously studied in pT1 bladder cancer by Krüger *et al* (2003) and muscle-invasive bladder disease by Korkolopoulou *et al* (2005), no data on Mcm2 expression in Ta/T1 BC have been published to date. Various cutoff levels have been reported for the evaluation of Mcm2 expression (Krüger *et al*, 2003; Korkolopoulou *et al*, 2005). A grouping using 20% steps proved to be most effective in our set; however, the exact classification in 20% steps is not easily reproducible between observers and discriminatory power was strongest between 0–20 and 81–100%. Since this grouping excludes many intermediate cases, we have chosen a cutoff level of 40%.

In our set of data a pathological expression of CK20 was not related to adverse clinical outcome. However, this finding has to be put into a careful perspective, as there are no data yet on a possible usefulness of CK20 in large sets of pT1 tumours. Furthermore, using tissue microarray (TMA) instead of large sections of whole tumour could lead to misjudgement of CK20 staining. In the present study, TMA cores with the relatively large diameter of 1.5 cm were constructed of most representative areas marked by one uropathologist (AH). This approach has been reported to be valid for the analysis of CK20 in Ta/T1 BC (van Oers *et al*, 2007).

Univariate Cox regression analysis showed grade, Ki67 and Mcm2 to be significantly related to recurrence rate. In a multivariate Cox regression model, Ki67 was no independent predictor of recurrence rate in contrast to grade and Mcm2.

Thus Mcm2 was a prognostic factor superior to Ki67, as it was independent and Mcm2 expression ⩾40% indicated a 5.81-fold increased hazard ratio.

Even taking confounding factors related to the limited size of the present series, the lack of follow-up information in roughly one-third of cases and the low number of pT1 cases into account and putting the findings into a very careful perspective, our data suggest Mcm2 expression to be superior to Ki67 in predicting the clinical course of Ta/T1 BC. We were able to define a valid threshold for the immunohistochemical evaluation of Mcm2, as 40% had a good discriminatory power for recurrence rate (*P*<0.001). In conclusion, our data warrant further evaluation of Mcm2 expression as a novel prognostic parameter for Ta/T1 BC.

## Figures and Tables

**Table 1:** Distribution of tumour recurrence rate

	**Recurrence**	***P-value***
*Mcm2 (*n=*69)*		*P*<0.001^*^
<40%	10/49 (20.4%)	
⩾40%	15/20 (75.0%)	
		
*CK20 (*n=*68)*		*P*=0.791 (NS)
Normal	10/27 (37.0%)	
Abnormal	15/41 (36.6%)	
		
*Ki67 (*n=*66)*		*P*=0.001^*^
<15%	11/46 (23.9%)	
⩾15%	13/20 (65.0%)	
*Grade (*n=*71)*		*P*<0.002^*^
G1	4/31 (12.9%)	
G2+G3	21/40 (56.3%)	
*Stage (*n=*71)*		*P*=0.656 (NS)
Ta	22/64 (34.4%)	
T1	3/7 (9.9%)	
*Tumour size (*n=*71)*		*P*=0.450 (NS)
<3 cm	63/71 (89%)	
⩾3 cm	8/71 (11%)	
*Multifocality (*n=*71)*		*P*=0.149 (NS)
Unifocal	51/71 (72%)	
Multifocal	20/71 (28%)	

NS=not significant; ^*^Significance at *P*<0.05.

**Table 2:** (a) Univariate and Cox regression analysis of Mcm2, Ki67, CK20, sex, age, grade, stage, tumour size and multifocality influencing tumour recurrence in patients with UCB and (b) Multivariate Cox regression analysis of Mcm2, Ki67, CK20, grade, stage, tumour size and multifocality influencing tumour recurrence in patients with UCB

(a)	**HR**	**95% CI**	***P*-value**
Ki67	3.39	1.50–7.68	**0.002**
CK20	1.37	0.58–3.24	0.481
Mcm2	4.75	2.12–10.65	**<0.001**
Stage	1.40	0.42–4.70	0.585
Grade	2.66	1.44–4.89	**<0.001**
Tumour size	6.87	3.13–10.89	**<0.001**
Multifocality	2.10	0.77–5.05	**0.005**
Sex	0.74	0.30–1.86	0.525
Age	1.01	0.98–1.04	0.814

(b) Stepwise reverse selection	**Adjusted HR**	**95% CI**	***P*-value**
Ki67	1.20	0.37–3.93	0.761
CK20	1.79	0.71–4.50	0.216
Mcm2	5.81	2.41–14.00	**<0.001**
Stage	1.64	0.36–7.42	0.521
Grade	2.37	1.24–4.51	**0.009**
Tumour size	6.87	3.05–11.72	**<0.001**
Multifocality	2.10	0.70–4.45	**0.003**

Abbreviations: CI=confidence interval, HR=Hazard ratio, UCB=urothelial carcinoma of the bladder. Bold values represent *P*-values <0.05.
